# Synthesis and Characterization of Erbium-Doped Silica Films Obtained by an Acid–Base-Catalyzed Sol–Gel Process

**DOI:** 10.3390/nano13091508

**Published:** 2023-04-28

**Authors:** Ali Abdullah, El Mostafa Benchafia, Daniel Choi, Sufian Abedrabbo

**Affiliations:** 1 Department of Physics, Khalifa University of Science and Technology, Abu Dhabi P.O. Box 127788, United Arab Emirates; ali.abdullah@ku.ac.ae (A.A.);; 2 Mechanical Engineering and Materials Science, Khalifa University of Science and Technology, Abu Dhabi P.O. Box 127788, United Arab Emirates; daniel.choi@ku.ac.ae

**Keywords:** erbium, sol-gel, thin-films, optical parameters, photoluminescence

## Abstract

Erbium-doped silica films were synthesized using a two-step sol–gel methodology that involved acid and base catalysts, with erbium concentration ranging from 0.2% to 6% and annealing temperatures varying from 500 °C to 900 °C. The photoluminescence spectra showed that the samples exhibiting efficient emission were annealed at 800 °C and 900 °C and doped with 3% and 6% erbium. The X-ray diffraction analysis revealed that the internal structure of the films was influenced by the different annealing temperatures and the doping concentrations. Samples with dominant 4f transitions were modelled. The results suggest that the proposed method is a promising approach for the synthesis of erbium-doped silica films with potential applications in optical devices.

## 1. Introduction

Due to their use in transceivers and laser systems, optical-gain materials have raised a great deal of scientific interest. Erbium-doped materials, for example, have a wide gain spectrum in fiber optics [1,2,3]. Owing to the clear potential for multiple transitions at various frequencies, due to their f-f energy states, these optical materials are widely used in visible to infrared solid-state lasers [4,5]. Furthermore, several practical optoelectronic applications have triggered tremendous scientists’ interests because of the special dielectric and photoluminescence properties of such rare earth oxides [6,7,8,9]. The lanthanides appear in the sixth row of the periodic table, where their 4f shell is half-filled. Normally, optical active rare-earth ions are created by releasing one 4f electron and two 6s electrons. These ions’ luminescence is mostly due to intra-4f or 5d–4f transitions. Since the 4f level is shielded by the 5d orbitals, the host material does not possess a strong effect on the intra-4f transitions but can have an effect on the Er-structures symmetry, Stark splits and the resulting recombination transitions’ peak position [10]. It should be noted that although intra-4f transitions for free ions are parity-banned, they are somewhat permitted once rare-earth ions are implanted in a host material due to the combination of contrary-parity wave functions. Consequently, they exhibit extended luminescence lifetimes and extremely weak oscillator strengths [11]. On the other hand, 5d–4f transitions are extremely affected by the neighboring ligands because 5d states are immediately accessible to the surrounding environment. Moreover, as 5d–4f transitions are parity-allowed, they offer extremely limited luminescence lifetimes and high oscillator strengths [11]. Up to date, improving the effectiveness of near-infrared photoluminescence from silica has been an ongoing research challenge to further exploit its potential in the optoelectronic industry. The low solubility of optically active erbium ions in diverse silicon materials is caused by the mismatch between the ionic radii and the matrix network’s high covalent bonding. This is the main cause of luminescence deterioration at the 4f erbium transition band [12]. Generally, rare-earth ions form precipitates at critical concentrations, leading to serious luminescence suppression either via ion–ion coupling or by the formation of an optically inactive phase [13,14]. This work was instigated by the strong success of bandgap efficiency enhancement from ordinary Cz-Si when interfaced with Er-doped sol–gel-based silica, which resulted from a joint effort between the principal of this group and international collaborators [15,16,17,18,19]. These studies focused mainly on relating the emission to radiative recombination across the Si-band [15,16,17,18] and on understanding the activation mode of Er as in reference [19]. In a subsequent study, the same group concentrated on understanding the pertaining effect of the dopant on the optical parameters utilizing the effective medium theorem [20]. All of these studies were limited to one doping concentration of erbium and to using a single catalyst for the sol–gel preparation. In this work, the novelty lies in investigating the effect of erbium doping concentration and subsequent annealing temperature on the near-infrared emissions at the 4f erbium transition band. This work is also unique in combining two catalysts for the sol–gel preparation, i.e., an acid followed by a base, with five different erbium concentrations, namely, 0.2%, 0.5%, 0.75%, 3% and 6%. As a matter of fact, this is the first time that anyone is reporting this unique combination of silica synthesized by two catalysts and the annealing of Er at various doping concentrations at five different temperatures. In this study, the temperature-dependent PL spectra are presented and were fitted to explain the mechanism behind the phonon-assisted energy transfer process and to show the dominant 4f transitions. In the subsequent sections of this study, a thorough structural analysis of the samples, conducted using X-ray diffraction techniques, is described. The phases detected by XRD were correlated with the photoluminescence data obtained at varying doping concentrations and annealing temperatures to gain a comprehensive understanding of the samples’ properties.

## 2. Experimental Details

The following materials were used as received: Sigma-Aldrich (Saint Louis, MI, USA)tetraethyl orthosilicate (TEOS, 99%) and Honeywell ethanol (EtOH, 99.8%). Sigma-Aldrich also supplied hydrochloric acid (HCl), ammonium hydroxide (NH_4_OH), and erbium (III) nitrate pentahydrate (Er(NO_3_)_3_·5H_2_O). Tetraethyl orthosilicate, known as TEOS, is used as a silicon alkoxide source to deposit silica. The sol–gel process was carried out in this work using two catalysts which were HCl and NH_4_OH. Erbium was incorporated into the silica matrix utilizing erbium (III) nitrate pentahydrate. The procedure of synthesizing the sol–gel films started with preparing the initial precursor by mixing TEOS, ethanol and water, followed by adding HCl. Ethanol here was used as a homogenizer, since TEOS is immiscible in water. In all experiments, the molar ratios of the components used in each experiment were kept constant as shown, i.e., TEOS: Water/Ethanol/Acid/Erbium = 1:4:10:0.003:x, where x = 0, 0.20, 0.50, 0.75, 3.0 and 6.0%. For 90 min, the mixture was refluxed and stirred at 70 °C and 300 rpm. After this process, the solution was divided into 5 equal volumes for erbium (III) nitrate pentahydrate to be added in the respective percentages mentioned above. Next, 0.1M NH_4_OH was added to each of the five solutions, and each of the 5 mixtures were then stirred at 300 rpm for 40 min. The sols were then dispensed through a syringe equipped with a 0.45 µm filter (PTFE Whatman (Maidstone, UK)), to allow the deposition onto (100) p-type square pieces of silicon wafers by spin coating at 1200 rpm for 30 s (Schwan Technologies (Marshall, MN, USA) provided the EZ-8 vacuum spin coater). The processed wafers were then baked at 120 °C in a conduction oven for one hour. Each coated substrate was then scribed into smaller pieces that were annealed at temperatures ranging from 500 °C to 900 °C. For simplicity, the dried but not annealed samples will be referred to as “as-deposited”. The refractive indices and film thickness measurements were obtained using a J. A. Woollam M-2000 XI Variable Angle Spectroscopic Ellipsometer (VASE) covering a range of wavelengths from UV to near-IR or from 210 nm to 1690 nm. The M-2000 utilizes a rotating compensator ellipsometer configuration and two CCD detectors. One detector was Si-CCD, from 210–1000 nm and with 1.5 nm resolution, and the other was InGaAs-CCD, from 1000–1690 nm with 3.5 nm resolution.

The crystallographic phases of erbium-doped silica films on silicon wafers were investigated through X-ray diffraction (XRD). A Bruker D2 Phaser XRD diffractometer with CuKα1 radiation at a wavelength of 1.54 nm was utilized in this study. The diffraction patterns were measured at room temperature in the 2θ range of 5–65° with 0.05° increments and a 0.75 s integration period for a total of a 15 min acquisition time. The geometry used was the classic Bragg–Brentano (BB) arrangement based on the theta-2-theta scan. The Profex [21] program was used to examine the XRD data and carry out and run the search and match algorithm for phase detection. Furthermore, the peaks observed were compared to literature data to determine what probable phases were present in the films [22]. Photoluminescence measurements were carried out at HORIBA labs. The room-temperature PL emission from the silicon bandgap and the Er^3+^ 4f transition band (~1535 nm) were obtained using a model Fluorolog Quanta Master (FL-QM) spectrofluorometer. A solid-state InGaAs detector was used for the NIR detection covering the range of 800–1700 nm. For excitation, a 980 nm laser diode that could vary the output power was used, thus allowing for a variation of the excitation power measurements which were performed on samples annealed at 800 °C and 900 °C with the doping Er concentrations of 3% and 6%.

## 3. Results and Discussion

### 3.1. Film Thickness and Refractive Index

In this work, a M-2000 XI J. A Wollam Variable Angle Spectroscopic Ellipsometer (VASE) with a monochromator and a rotating analyzer was used to obtain the films’ thickness and optical constants measurements. The ellipsometry data analysis was performed using the Wollam CompleteEase software; the Sellmeier fitting model, which relates the refractive index to the wavelength, was used for an excellent agreement with the processed samples. We used a three-layer model consisting of the substrate, the thin oxide layer and the film under investigation to recuperate the optical constants. The fitting quality was measured by the root-mean-squared error (MSE) between the measured data and the generated models, and in most cases, the MSE was low. In a few instances, we reduced the number of measurement angles from 7 to 4 or 3 to obtain a better fit. To analyze the spectroscopic ellipsometry data experimentally collected using the M-2000 XI ellipsometer—Ψ and Δ of the polarized reflected light—a mathematical model was required to accurately interpret the results. The refractive indices and thicknesses of the deposited films were deconvoluted from the modelled data. Root-mean-squared errors (MSE) of values, approved by the CompleteEase and M 2000 XI system (J.A. Woollam), below 10 were observed, eliminating the need for other fitting models. This model is composed of a set of adjustable parameters that are used to match the theoretical spectra generated by the model to the experimental data.

**Figure 1:**
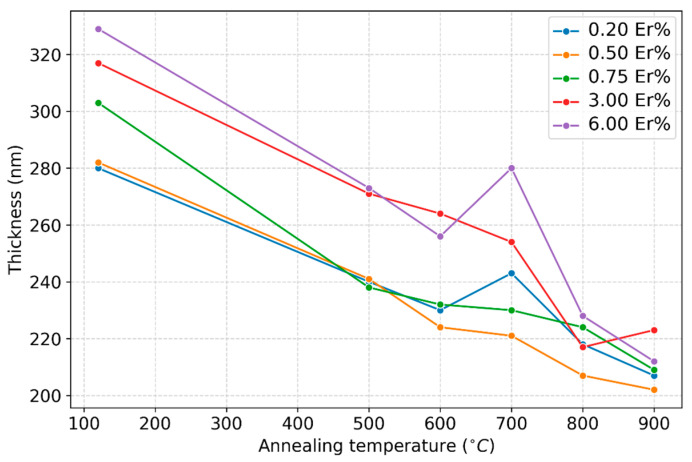
Variable doped-film thickness at annealing temperatures in the 500–900 °C range.

The Sellmeier relationship is a superior model for transparent materials or lowly doped films, particularly for low MSE values. The Sellmeier equation is written in its most generic form as:

(1) $$n^{2}-1=\underset{i}{\sum }\frac{A_{i\;}\lambda^{2}}{\lambda^{2}-\lambda_{i}}$$

where $A_{i}$ and $\lambda_{i}$ are Sellmeier coefficients established empirically. The model has the extra advantage of maintaining Kramers–Kronig consistency, assuring a physical form for the resultant refraction index. To adjust for UV and IR absorption, CompleteEASE employs a two-term Sellmeier model. The built-in equation of the refraction index in the software is represented as follows:

(2) $$n\left(\lambda \right)={\left(1+\frac{A\lambda^{2}}{\lambda^{2}-B^{2}}-E\lambda^{2}\right)}^{\frac{1}{2}}$$

where *A* is the amplitude, *B* is the center energy, and *E* is the position of a pole in the infrared region. Table 1 below shows the coefficients for the obtained film doped with 6% Er at 900 °C:

The thickness of the synthesized samples is presented in Figure 1. The thickness of the as-deposited films was found to increase with the Er doping concentration. In order to understand the relation between Er concentration and film thickness, one needs to first note that the source of Er was erbium nitrate pentahydrate. Increasing the concentration of erbium in the silica matrix required higher amounts of erbium nitrate, which eventually lowered the pH of the solution, making it more acidic, as it dissociated in water releasing (Er^3+^) and nitrate ions (NO^3−^). The nitrate ions hydrolyzed in water to produce nitric acid (HNO_3_) and hydroxide ions (OH^−^). The hydroxide ions then reacted with the metal alkoxide precursor to initiate the sol–gel reaction, while the nitric acid increased the acidity of the sol, lowering its pH. As a result, in the sol–gel process, hydrolysis became more dominant than condensation. The lower level of condensation resulted in a gel network that was more porous and weakly adherent. However, rare-earth ions with a relatively large radius, ranging from 0.868 to 1.36 Å [23], are able to compensate for this effect by inducing larger d spacing and lattice parameters of the species formed. This interplay between the increase in porosity and the incorporation of larger dopants resulted in an enhancement of the film thickness at higher erbium concentrations. Furthermore, a higher concentration of optically active Er sites within the silica matrix led to a higher photoluminescence efficiency at higher erbium concentrations. Upon annealing, the thickness of all processed films, regardless of the doping concentration, decreased as a consequence of the removal of the organic groups and residues during the sol–gel process. The shrinking taking place persisted up to 600 °C. Between 600 °C and 700 °C, an unexpected increase in thickness was clearly noticeable for all samples.

**Figure 2:**
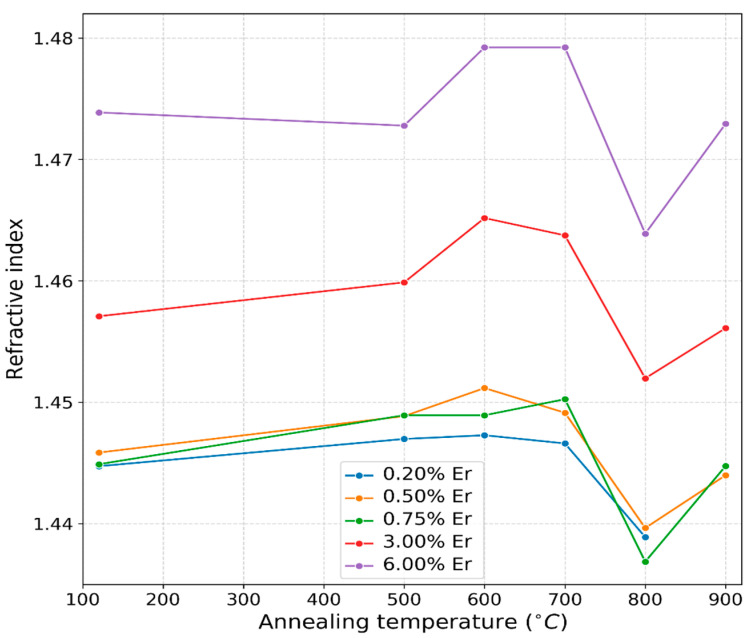
Refractive indices variation at annealing temperatures in the 500–900 °C range under different doping concentrations. The refractive index measurements were performed at the wavelength of 632.8 nm.

As is well known, sol–gel-coated films possess a high content of OH^−^ anions that are known to diffuse more rapidly than O as in wet oxidation vs. dry oxidation, and the onset of oxidation of Si beyond the native oxide is at or above 600 °C [24]. It was, therefore, anticipated that at 700 °C, the top part of the Si-substrate beneath the film coatings would be consumed via wet oxidation, forming thermal oxide, thus increasing the thickness of the overall dielectric layer measured by the M-2000 XI ellipsometer. Beyond 700 °C, the shrinkage process of the silica coatings continued, and voids and pores continued shrinking, while the layer resulting from the thermal oxidation of the Si substrate became a barrier for the further consumption of the Si substrate, besides the fact that less OH anions were available in the annealed films to diffuse and to form thermally grown SiO_2_, forcing the films’ thickness to shrink further. The behavior of the film thickness with respect to heat treatment was further reflected in the extracted optical constants and light emission, as discussed in the remaining sections. The 700 °C temperature was thus believed to be a threshold where a drastic change took place for the erbium silica films synthesized using this sol–gel process. The refractive index measurements acquired by ellipsometry at the 632.8 nm wavelength are shown in Figure 2. Irrespective of erbium content or annealing temperature, all of the processed films exhibited a refractive around 1.4, which is typical for silica glass. The refractive index of the film doped with 6% Er at 600 °C and 700 °C was 1.478 and was the highest among those of all tested samples. In general, the refractive index of the doped samples increased as the erbium level increased. The samples could be divided in two groups depending on the doping concentration. It was expected that the higher Er concentration increased the films optical parameters appreciably [20], and this is clearly shown for erbium doping concentrations of 3% and 6%. The Sellmeier model utilized did not show the same results for lower doping concentrations (i.e., 0.2, 0.5 and 0.75%).

### 3.2. Structural Analysis (X-ray Diffraction)

**Figure 3:**
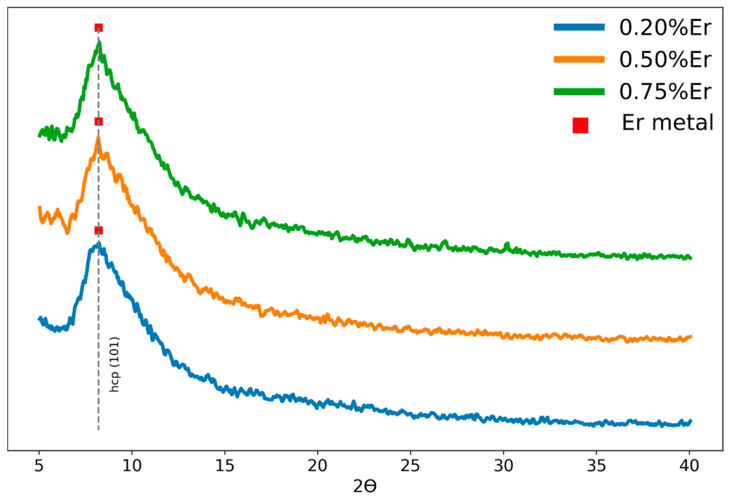
XRD pattern for 0.2%, 0.5 and 0.75% erbium-doped films on a Si substrate. The dashed line represents the (101) plane with the hcp space group [28].

**Figure 4:**
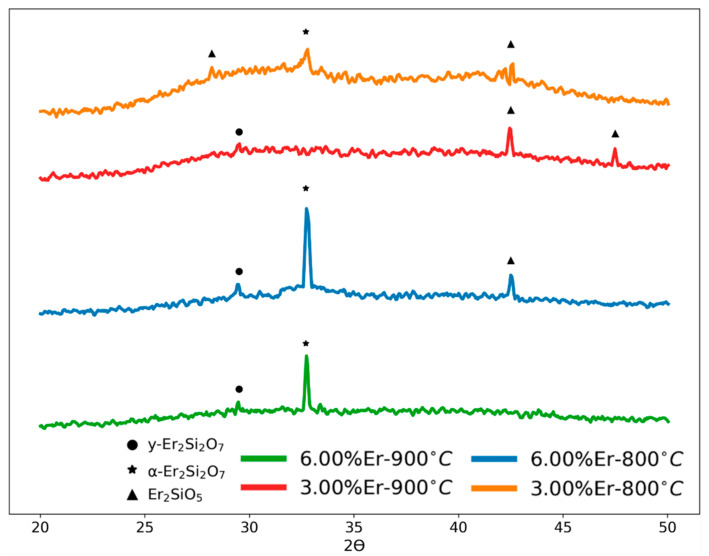
XRD pattern for 3% and 6% Er-doped silica at the annealing temperatures of 800 °C and 900 °C.

XRD analysis was performed to probe any ordered structures involving silicate phases resulting from the annealing process which was conducted in the range from 500 to 900 °C. The raw data were analyzed using the Rietveld [25] method with the Profex [26] code. Figure 3 presents the XRD patterns of three Er-doped films (0.2%, 0.5% and 0.75% Er) deposited on the Si (100) substrate and annealed at 800 °C. All films were amorphous, having a diffuse peak within the angle range from 6° to 20°, according to the XRD patterns [27]. A preferred orientation along the (101) plane with the hcp space group was identified, indicating the potential of having Er metal clusters (as indicated in the erbium XRD spectra [28]). This might have a significant relationship with the PL data of these samples, which will be explored in the next part. The Si (100) substrate overwhelmed the XRD diffraction patterns with its prominent peak of about 69.2°. The XRD data were thus truncated to the (5–50°) 2Ɵ angles in order to facilitate the phase detection. The combination of higher doping concentrations and annealing temperatures had a direct influence on the patterns and the intensity of the peaks, as seen in Figure 4. By introducing the elements Si, O, Er into the “search and match” algorithm of the Profex package, the resolved peaks related to the Er–Si–O phases within the silica matrix were predominantly Er_2_Si_2_O_7_ and Er_2_SiO_5_. It is worthwhile to share that the samples with higher Er doping concentrations did also exhibit the same feature at about 2θ ~ 8.2° associated with the hcp-Er metal cluster, but we omitted this feature from Figure 4 to allow for analyzing other smaller, resolved peaks with clarity.

After annealing at temperatures ranging from 900 °C to 1150 °C, Gao et al. [22] showed four distinct erbium silicate polymorphs in the Er–Si–O films: Er_2_SiO_5_, y-Er_2_Si_2_O_7_, α-Er_2_Si_2_O_7_ and γ-Er_2_Si_2_O_7_, whereas annealing the Er silicates at higher temperatures led to more stable polymorphs. Moreover, at 1150 °C, the Er_2_SiO_5_ phase was found to be entirely converted into y-Er_2_Si_2_O_7_. In our case, as depicted in Figure 4, the deposited films exhibited varying intensities at various angles, which corresponded to different phases of the material. Specifically, the 33.3° angle indicates the presence of the triclinic-Er_2_Si_2_O_7_ phase, the 29.3° angle corresponds to the monoclinic-Er_2_Si_2_O_7_ phase, and both the 42.2° and the 47.6° angles represent the monoclinic-Er_2_SiO_5_ phase. In order to put things in perspective, the results in Figure 4 for y-Er_2_Si_2_O_7_ at 2θ = 33.3° for the various spectra were compared. For the 6% Er-containing samples, the peak for the samples annealed at 800 °C showed a 19% higher intensity compared to that for samples annealed at 900 °C. It was also found that for the samples annealed at 800 °C, the peak of the sample containing 6% Er was 73% higher that the peak of the 3% Er sample. The photoluminescence properties of these polymorphs exhibited a significant degree of diversity. The presence of these phases will be considered to further explain the photoluminescence behavior in the following section.

### 3.3. Photoluminescence Measurements

Understanding how to regulate the processes to synthesize Er-doped films while avoiding luminescence quenching is required to obtain specific samples with effective emission using erbium-doped silicon. The focus and discussion in this section will be on emission resulting from transitions within the 4f transition of Er.

#### Erbium Emission at the 4f Transition Band

**Figure 5:**
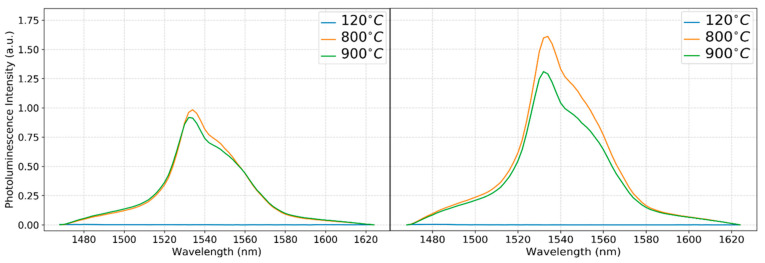
Intensity variation with the wavelength for as-deposited and multiple-annealed 3.00% and 6.00% Er-doped films.

Figure 5 shows the PL intensities of erbium-doped silica deposited on Si substrates as a function of the wavelength. The processed samples were annealed at temperatures ranging from 500 °C to 900 °C. The Er^3+^ cations are known to be optically activated when annealed at 800 °C or above. Only samples with Er concentrations of 3% and 6% that were annealed at 800 °C and 900 °C are shown, due to the inefficient emission from lower doping concentrations that will be then discussed in light of the XRD results.

The major cause of this quenching might be the ion–ion relationship, which causes erbium clustering, which prevailed in the lower-Er-concentration-doped samples. Despite having a similar peak to that observed at higher Er doping concentrations, the abundant Er atoms that were distributed across the silica ensured a large number of optically active Er. The XRD examination consistently showed an intensity at ~8.2°, which corresponds to erbium’s inactive optical phase [28], indicating that erbium clustering was a key cause of quenching. In addition, PL quenching is typically linked to erbium ion cross-relaxation processes in clustering Er–O–Er bonds [29]. Another reason for luminescence quenching at this band could be the energy transfer processes owing to the interactions between erbium ions (Er^3+^) and hydroxyl (OH^−^) groups at high erbium doping concentrations [30,31,32]. Since the ammonia base was utilized as a catalyst in our recipe preparation, the concentration of hydroxyl ions was high, increasing the likelihood of luminescence quenching at lower Er concentrations, given that most activated Er ions might be surrounded by OH anions. All emissions observed were at high doping levels and an annealing temperature of 800 °C or 900 °C. With 6% erbium doping, the highest emission was achieved at 800 °C. Then, by the given sequence, (900 °C-6% Er) → (800 °C-3% Er) → (900 °C-3% Er), less emission was observed at the 4f transition band. At these doping concentrations, there existed an abundance of activated Er ions that survived the OH interaction, resulting in discernible 4f radiative recombination. The varying diffraction peaks obtained from the XRD examination of these samples, as shown in Figure 4, indicated the existence of several Er silicate polymorphs. For example, at 900 °C, 3%-E), we observed peaks referring to the monoclinic-Er_2_SiO_5_ phase, which had a relatively lower active luminescence phase compared to other Er_2_Si_2_O_7_ phases. The spectra representing the 800 °C, 6% Er condition, on the other hand, showed intensities that indicated the presence of two Er_2_Si_2_O_7_ phases that had a higher luminescence activity [22]. In addition, it can be noticed that the PL intensity at 800 °C for both 3% and 6% erbium-doped samples was higher compared to the emission measured from the 900 °C samples. To elaborate further on the relation between the phases and the PL intensities observed, we resort again to the work of Gao et al. [22]

Many materials for the fabrication of active and passive media for laser technology, photonic devices, and telecommunications are produced by doping silica, notably, by rare-earth metals [33,34,35]. The crystalline and amorphous environments have received particular attention because they can show high-intensity emission when subjected to extrinsic influences [36].

**Figure 6:**
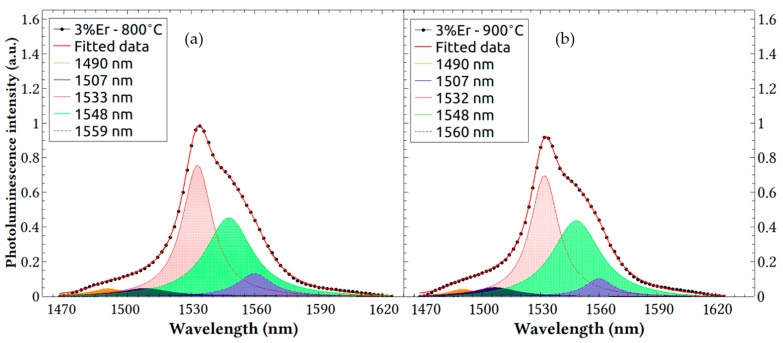
Lorentzian band deconvolution for PL spectra of silica doped with 3% Er at annealing temperature of (**a**) 800 °C and (**b**) 900 °C.

**Figure 7:**
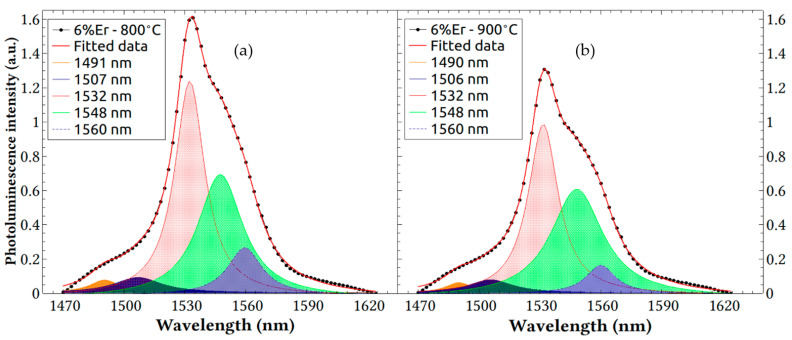
Lorentzian band deconvolution for PL spectra of silica doped with 6% Er at annealing temperatures of (**a**) 800 °C and (**b**) 900 °C.

The PL spectra revealed that the silica host matrix induced an odd-parity character in perturbed Er 4f wave functions. This enabled the optical activity observed in Figure 5 and allowed for radiative transitions to be observed at various energy levels, as will be demonstrated in the modelling below. Silica host formed by sol–gel techniques are generally amorphous and possess a variety of pores, implying that the Er ions may occupy more than one atomic site (known as activator centers) in the host matrix. Each site possesses a different symmetry and, hence, different crystal fields, resulting in yield randomization of the Stark splitting effect of the first excited state of ^4^I_13/2_ and ground state ^4^I_15/2_ levels of Er 4f. This entails inhomogeneous broadening and leads to an emission band spanning the wavelengths from 1480 to 1560 nm [37,38]. We applied various analysis methods to model the data and adopted a Lorentzian fit model that yielded the best coefficient of determination (COD) value (R^2^), greater than 0.999, indicating a high level of correlation for the modeled data when fitting using five peaks. The results of the fitting analysis of the observed spectra in Figure 5 are presented in Figure 6a,b and Figure 7a,b, where the original spectrum is represented by the black line, and the data are modeled by superposition of the spectral transitions at multiple wavelengths, represented by the red line. The deconvolution was performed on QtiPlot software.

The fitted peaks not only yielded a good fit, but also resulted in a set of peaks that were traceable to known and possible transitions from the first excited state to the ground state within the 4f splitting. After setting the initial five guesses, the model was allowed to vary the parameters in the superposition equation until obtaining the best fit. We performed a Lorentzian fitting in order to obtain the peak positions and linewidths accurately. This fitting methodology was applied in different previous works [39,40]. The wavelength, the area under the fitted curves (AUFC), the relative intensity and the FWHM for each peak that varied with the concentration of Er and the annealing temperature are listed in Table 2 and Table 3.

Figure 6a,b presents the experimental photoluminescence spectra and the modelled data for the films containing 3% Er. Table 2 summarizes the fitted peaks. As can be seen, the deconvoluted PL spectra showed two dominant emission peaks at 1532 nm and 1548 nm. Moreover, three shoulder peaks are shown at 1490 nm, 1506 nm and 1560 nm. As was explained earlier, the 4f transitions possess some immunity from the host material and the overall symmetry, although the dominant peaks or preferred recombination transitions can vary. As a manifestation to this fact, we would like to bring the attention to the fact that the samples in this work were processed using sol–gel techniques, and that the transitions indicated on page 238 of reference [41] were for Er-doped aluminosilicate, while the ones in the work of Abedrabbo et al. in references [42,43] were for ion beam mixing of Er, Si and O and for Er, Si, O and Ge, and all of them shared similar transitions. The ion beam mixing effort of this group started earlier without Er impurity centers as in references [44,45] and was consolidated with rare-earth inclusions. As a matter of fact, it is noteworthy to state that the transitions approximately corresponded to 1480 nm, 1500 nm, 1516 nm, 1535 nm and 1550 nm, and 1487 nm, 1511 nm, 1538 nm and 1551, respectively. This work shares one dominant transition around 1532 nm which was tied to a transition from energy level 6644 cm^−1^ from I_13/2_ to level 130 cm^−1^ in the I_15/2_ ground state. Furthermore, all the other peak wavelengths could be traced to known transitions or small perturbations from the transitions reported in reference [41] as follows: the 1548 nm wavelength corresponds to transition F7 from 6644 cm^−1^ to 201 cm^−1^; the 1490 nm one corresponds to transition F8 from 6711 cm^−1^ to 0 cm^−1^; the 1506 nm one corresponds to transition F4 from 6644 cm^−1^ to 0 cm^−1^; and 1560 corresponds to F3 transition from 6548 cm^−1^ to 130 cm^−1^. Since the luminescence of Er ions is broadband luminescence, the luminescence intensity is characterized by the integral of the luminescence area. With the increase in the Er concentration, the integral of the luminescence area at ~1532 nm (Peak 3) increased. When the annealing temperature was 900 °C, the integral of the luminescence area decreased for both Er concentrations (3% and 6%).

The same analysis, shown in Figure 7a,b, was performed for the experimental photoluminescence data spectra for the 6% Er-doped processed films. Again, it is noticed that the fitted data well correlated with the experimental data and that the prevailing five peaks shared the same wavelength positions. This again revealed that, in addition to the fact that the host or co-dopants had little effect on the 4f transitions, the Er doping concentration that was doubled from 3% to 6% did not cause dramatic shifts in the resolved peaks and discernible changes in optical activity, despite the higher chances for Er–Er segregation. One can notice, however, that the 6% Er concentration exhibited higher overall emission superposition of the resolved peaks as well as higher individual peaks and a larger area under the curve than the 3% concentration shown in Figure 6, proving that the followed process yielded even more highly optically active Er centers.

**Figure 8:**
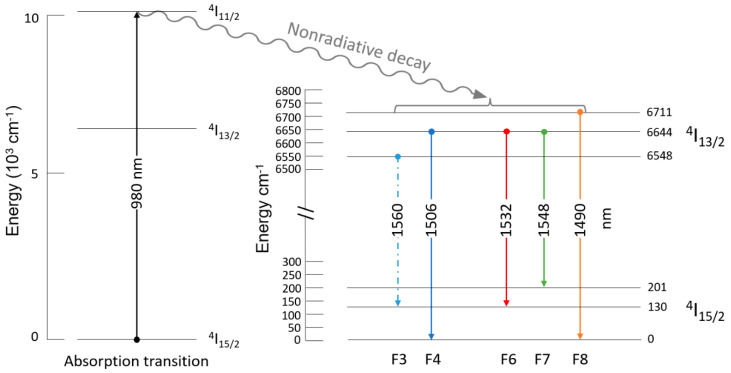
Energy level diagram showing stark transitions corresponding to absorption and emission in Er–SiO_2_ with corresponding peak wavelengths.

In summary, as the erbium concentration increased, the areas of peak 2 and peak 3 increased, and their FWHM changed little; so, the intensity peaks became higher. The excitation was performed by a laser at 980 nm wavelength, which corresponds to the ^4^I_15/2_ → ^4^I_11/2_ transition, whereas the PL emission band was due to the ^4^I_13/2_ → ^4^I_15/2_ transition in Er^3+^ ions. The PL intensity rose as the Er content increased. The amount of Er or other elements by itself in the silica matrix had no effect on where the luminescence maximum occurred at 1532 nm. For achieving a better visibility of the optical transitions briefed above, Figure 8 represents a summary of the absorption process using 980 nm as the excitation source to excite optically active Er to the I_11/2_ level, followed by a quick decay into the metastable level of I_13/2_ before returning to ground state of I_15/2_.

## 4. Conclusions

In this study, Er-doped silica films were processed and deposited on crystalline Si in order to examine their light emission in the 4f transition band. The films were thoroughly characterized, and the process parameters were adjusted to achieve optimal film uniformity. The optimized protocol produced homogeneous and crack-free films. Erbium-doped silica films were deposited on silicon substrates and annealed at various temperatures ranging from 500 °C to 900 °C, using an acid–base catalyst. The study found that the samples annealed at 800 °C exhibited the highest level of light emission in the erbium transition band at high erbium doping. Overall, the study demonstrates that Er-doped silica films have the potential to be utilized in optoelectronic applications due to their high light emission efficiency.

## Figures and Tables

**Table 1:** Coefficients of the Sellmeier equation.

Coefficient	Value
A	0.215 ± 0.00619
B	0.16674 ± 0.001414
E	0.943 ± 00048588

**Table 2:** Area under the curve, main peak position, FWMH and relative peak intensity of 3% Er-doped at annealing temperatures of 800 and 900 °C.

Sample		3%—800 °C					3%—900 °C			
Peak NO.	P1	P2	P3	P4	P5	P1	P2	P3	P4	P5
AUFC	1.926	1.876	17.87	15.69	3.535	1.051	2.297	21.85	19.61	2.823
Peak position (nm)	1490	1507	1533	1548	1559	1490	1507	1532	1548	1560
FWMH (nm)	17.19	27.004	17.23	26.76	19.36	16.55	27.52	17.67	30.41	17.31
Peak intensity (a.u.)	0.0440	0.0463	0.758	0.452	0.131	0.0404	0.0532	0.707	0.457	0.104

**Table 3:** Area under the curve, main peak position, FWMH and relative peak intensity of 6% Er doped at annealing temperatures of 800 and 900 °C.

Sample		6%—800 °C					6%—900 °C			
Peak NO.	P1	P2	P3	P4	P5	P1	P2	P3	P4	P5
AUFC	2.003	4.078	35.56	30.82	8.955	1.553	3.357	28.31	30.71	4.637
Peak position (nm)	1491	1507	1532	1548	1560	1490	1506	1532	1548	1560
FWMH (nm)	17.39	28.39	17.98	27.06	20.47	16.11	26.96	18.06	31.32	17.67
Peak intensity (a.u.)	0.073	0.091	1.259	0.724	0.278	0.061	0.0792	0.998	0.634	0.167

## Data Availability

The data presented in this study are available on request from the corresponding author.
